# Supplementary material for: Computational design of non-porous pH-responsive antibody nanoparticles
Source: Nat Struct Mol Biol. 2024 May 9;31(9):1404–12. doi: 10.1038/s41594-024-01288-5 (PMC11402598; doi:10.1038/s41594-024-01288-5)
Supplement: Supplementary file 1 — Supplementary Tables 1–9 and Figs. 1–4 containing uncropped gels for Extended Data figures. [file 41594_2024_1288_MOESM1_ESM.pdf]

nature structural & molecular biology

Article

https://doi.org/10.1038/s41594-024-01288-5

# Computational design of non-porous pH-responsive antibody nanoparticles

In the format provided by the authors and unedited

# Supplementary Tables

### Supplementary Table 1: Amino acid sequences of assembling O432-17 designs

| Design name | Sequence |
|---|---|
| O432-17-C3 | MSEEKIEKLLEELTASTAELKRATASLRAITEELKKNPSEDALVEHNRAIVEHNAIIVENNRIIATVLLAIVAAIATNEATLAADKAKEAGASEVAKLAKKVLEEAEELAKENDSEEALKVVKAIADAAKAAAEAAREGKTEVAKLALKVLEEAIELAKENRSEEALKVVREIARAALAAAQAAEEGKTEVAKLALKVLEEAIELAKENRSEEALKVVREIARAALAAAQAAEEEGKTEVAKLALEVLEQAIEAAKLQRSERALEMVREIARAALEAARNAEGGRSDRARAILASLKVSIIVVKLKSSGTSEEEILRIVLKIIKELRKTAKESGQSASYIATMEAEIVKAIDYALDLSGTSGSWSGLEHHHHHH |
| O432-17-C4 | MFNKDQQSAFYEILNMPNLNEALRNGFIQLLKDDPSKSEVILTAALIAAKLSEDIRTLKESGSSYEEIAERVARAVALLVALLKTNGVSEDEIALAVALIISAVIQTLKESGSSYEVIAEIVARIVAEIVEALKRSGTSEDEIAEIVARVISEVIRTLKESGSSYEVIAEIVARIVAEIVEALKRSGTSEDEIAKIVARVIAEVLRTLKESGSSEEVIKEIVARIITEIKEALKRSGTSEDEIELITLMIEAALEIAKLKSSGSEYEEIAEDVARRIAELVEKLKRDGTSAVEIAKIVAAIISAVIAMLKASGSSYEVIAEIVARIVAEIVEALKRSGTSAAIIALIVALVISEVIRTLKESGSSFEVILEIVIRIVLEIIEALKRSGTSEQDVMLIVMAVLLVVLATLQLSGSGSWSGLEHHHHHH |
| Negatively charged variants | |
| O432-17(-)_2HIS-C3 | MSEEKIEKLLEELTASTAELKRATASLRAITEELKKNPSEDALVEHNRAIVEHNAIIVENNRIIATVLLAIVAAIATNEATLAADKAKEAGASEVAELAKEVLEEAEELAKENDSEEALKVVKAIADAAKAAAEAAREGKTEVAELALKVLEEAIELAKENRSEEALKVVREIARAALAAAQAAEEGKTEVAELALEVLEEAIELAKENRSEEALKVVREIARAALAAAQAAEEGKTEVAELALEVLEQAIEAAKLQRSERALEMVREIARAALEAARNAEGGRSDRARAILASLKVSIIVVKLKSSGTSEEEILRIVLKIIKELRKEAKEEGQSASYIATMEAEIVKAIDYALDLSGTSGSWSGLEHHHHHH |
| O432-17(-)_3HIS_I57V_L75A-C3 | MSEEKIEKLLEELTAATAELKRATASLRAITEELKKNPSEDALVEHNRAIVEHNAIVVEHNRIIATVLLAIVAAAATNEATLAADKAKEAGASEVAELAKEVLEEAEELAKENDSEEALKVVKAIADAAKAAAEAAREGKTEVAELALKVLEEAIELAKENRSEEALKVVREIARAALAAAQAAEEGKTEVAELALEVLEEAIELAKENRSEEALKVVREIARAALAAAQAAEEGKTEVAELALEVLEQAIEAAKLQRSERALEMVREIARAALEAARNAEGGRSDRARAILASLKVSIIVVKLKSSGTSEEEILRIVLKIIKELRKEAKEEGQSASYIATMEAEIVKAIDYALDLSGCSGSWSGLEHHHHHH |
| O432-17(-)_0HIS-C3 | MSEEKIEKLLEELTASTAELKRSTASLRASTEELKKNPSEDALVENNRLIVENNAIIVENNRIIATVLLAIVAAIATNEATLAADKAKEAGASEVAELAKEVLEEAEELAKENDSEEALKVVKAIADAAKAAAEAAREGKTEVAELALKVLEEAIELAKENRSEEALKVVREIARAALAAAQAAEEGKTEVAELALEVLEEAIELAKENRSEEALKVVREIARAALAAAQAAEEGKTEVAELALEVLEQAIEAAKLQRSERALEMVREIARAALEAARNAEGGRSDRARAILASLKVSIIVVKLKSSGTSEEEILRIVLKIIKELRKEAKEEGQSAS |

| | |
|---|---|
| | YIATMEAEIVKAIDYALDLSGCSGSWSGLEHHHHHH |
| O432-17(-)_3HIS_I57V-C3 | MSEEKIEKLLEELTAATAELKRATASLRAITEELKKNPSEDALVEHNRAIVEHNAIVVEHNRIIATVLLAIVAAIATNEATLAADKAKEAGASEVAELAKEVLEEAEELAKENDSEEALKVVKAIADAAKAAAEAAREGKTEVAELALKVLEEAIELAKENRSEEALKVVREIARAALAAAQAAEEGKTEVAELALEVLEEAIELAKENRSEEALKVVREIARAALAAAQAAEEGKTEVAELALEVLEQAIEAAKLQRSERALEMVREIARAALEAARNAEGGRSDRARAILASLKVSIIVVKLKSSGTSEEEILRIVLKIIKELRKEAKEEGQSASYIATMEAEIVKAIDYALDLSGCSGSWSGLEHHHHHH |
| O432-17(-)_3HIS_L75A-C3 | MSEEKIEKLLEELTAATAELKRATASLRAITEELKKNPSEDALVEHNRAIVEHNAIIVEHNRIIATVLLAIVAAAATNEATLAADKAKEAGASEVAELAKEVLEEAEELAKENDSEEALKVVKAIADAAKAAAEAAREGKTEVAELALKVLEEAIELAKENRSEEALKVVREIARAALAAAQAAEEGKTEVAELALEVLEEAIELAKENRSEEALKVVREIARAALAAAQAAEEGKTEVAELALEVLEQAIEAAKLQRSERALEMVREIARAALEAARNAEGGRSDRARAILASLKVSIIVVKLKSSGTSEEEILRIVLKIIKELRKEAKEEGQSASYIATMEAEIVKAIDYALDLSGCSGSWSGLEHHHHHH |
| Positively charged variants | |
| O432-17(+)_2HIS-C3 | MSEEKIEKLLEELTASTAELKRATASLRAITEELKKNPSEDALVEHNRAIVEHNAIIVENNRIIATVLLAIVAAIATNEATLAADKAKEAGASEVAKLAKKVLKQAEQLAKENDSEEALKVVKAIADAAKAAAEAAREGKTEVAKLALKVLANAIKLAKENRSEEALKVVREIARAALAAAQAAEEGKTEVARLALKVLQNAIQLAKENRSEEALKVVREIARAALAAAQAAEEGKTEVAKRALKVLQQAIQAAKLQRSERALEMVREIARAALEAARNAEGGRSDRARAILASLQVSIIVVKLKSSGTSEEEILRKVLKIIKELRKKAKEQGQSASYIATMEAEIVKAIDYALDLSGTSGSWSGLEHHHHHH |
| O432-17(+)_3HIS_I57V-C3 | MSEEKIEKLLEELTAATAELKRATASLRAITEELKKNPSEDALVEHNRAIVEHNAIVVEHNRIIATVLLAIVAAIATNEATLAADKAKEAGASEVAKLAKKVLKQAEQLAKENDSEEALKVVKAIADAAKAAAEAAREGKTEVAKLALKVLANAIKLAKENRSEEALKVVREIARAALAAAQAAEEGKTEVARLALKVLQNAIQLAKENRSEEALKVVREIARAALAAAQAAEEGKTEVAKRALKVLQQAIQAAKLQRSERALEMVREIARAALEAARNAEGGRSDRARAILASLQVSIIVVKLKSSGTSEEEILRKVLKIIKELRKKAKEQGQSASYIATMEAEIVKAIDYALDLSGCSGSWSGLEHHHHHH |
| O432-17(+)_3HIS_L75A-C3 | MSEEKIEKLLEELTAATAELKRATASLRAITEELKKNPSEDALVEHNRAIVEHNAIIVEHNRIIATVLLAIVAAAATNEATLAADKAKEAGASEVAKLAKKVLKQAEQLAKENDSEEALKVVKAIADAAKAAAEAAREGKTEVAKLALKVLANAIKLAKENRSEEALKVVREIARAALAAAQAAEEGKTEVARLALKVLQNAIQLAKENRSEEALKVVREIARAALAAAQAAEEGKTEVAKRALKVLQQAIQAAKLQRSERALEMVREIARAALEAARNAEGGRSDRARAILASLQVSIIVVKLKSSGTSEEEILRKVLKIIKELRKKAKEQGQSASYIATMEAEIVKAIDYALDLSGCSGSWSGLEHHHHHH |
| O432-17(+)_3HIS_I57V_L75A-C3 | MSEEKIEKLLEELTAATAELKRATASLRAITEELKKNPSEDALVEHNRAIVEHNAIVVEHNRIIATVLLAIVAAAATNEATLAADKAKEAGASEVAKLAKKVLKQAEQLAKENDSEEALKVVKAIADAAKAAAEAAREGKTEVAKLALKVLANAIKLAKENRSEEALKVVREIARAALAAAQAAEEGKTEVARLALKVLQNAIQLAKENRSEEALKVVREIARAALAAAQAAEEGKTEVAKRALKVLQQAIQAAKLQRSERALEMVREIARAALEAARNAEGGRSDRARAILASLQVSIIVVKLKSSGTSEEEILRKVLKIIKELRKKAKEQGQSASYIATMEAEIVKAIDYALDLSGCSGSWSGLEHHHHHH |

| | |
|---|---|
| O432-17(+)_0HIS-C3 | MSEEKIEKLLEELTASTAELKRSTASLRASTEELKKNPSEDALVENNRLIVENNAII VENNRIIATVLLAIVAAIATNEATLAADKAKEAGASEVAKLAKKVLKQAEQLAKEND SEEALKVVKAIADAAKAAAEAAREGKTEVAKLALKVLANAIKLAKENRSEEALKV VREIARAALAAAQAAEEGKTEVARLALKVLQNAIQLAKENRSEEALKVVREIARA ALAAAQAAEEGKTEVAKRALKVLQQAIQAAKLQRSERALEMVREIARAALEAAR NAEGGRSDRARAILASLQVSIIVVKLKSSGTSEEEILRKVLKIIKELRKKAKEQGQ SASYIATMEAEIVKAIDYALDLSGCSGSWSGLEHHHHHH |

**Supplementary Table 2: Amino acid sequences of hIgG1-Fc, Fc-fusions, and IgGs produced and used to assemble O432-17 designs**

| Design name | Sequence |
|---|---|
| hIgG1-Fc | EPKSSDKTHTCPPCPAPELLGGPSVFLFPPKPKDTLMISRTPEVTCVVVDVSHE DPEVKFNWYVDGVEVHNAKTKPREEQYNSTYRVVSVLTVLHQDWLNGKEYKC KVSNKALPAPIEKTISKAKGQPREPQVYTLPPSRDELTKNQVSLTCLVKGFYPSDI AVEWESNGQPENNYKTTPPVLDSDGSFFLYSKLTVDKSRWQQGNVFSCSVMH EALHNHYTQKSLSLSPGK |
| sfGFP-Fc | SRATMETDTLLLWVLLLWVPGSTGHHHHHHGGSENLYFQGGSSKGEELFTGVV PILVELDGDVNGHKFSVRGEGEGDATNGKLTLKFICTTGKLPVPWPTLVTTLTYG VQCFSRYPDHMKRHDFFKSAMPEGYVQERTISFKDDGTYKTRAEVKFEGDTLV NRIELKGIDFKEDGNILGHKLEYNFNSHNVYITADKQKNGIKANFKIRHNVEDGSV QLADHYQQNTPIGDGPVLLPDNHYLSTQSVLSKDPNEKRDHMVLLEFVTAAGIT HGMDELYKGGSGSEPKSSDKTHTCPPCPAPELLGGPSVFLFPPKPKDTLMISRT PEVTCVVVDVSHEDPEVKFNWYVDGVEVHNAKTKPREEQYNSTYRVVSVLTVL HQDWLNGKEYKCKVSNKALPAPIEKTISKAKGQPREPQVYTLPPSRDELTKNQV SLTCLVKGFYPSDIAVEWESNGQPENNYKTTPPVLDSDGSFFLYSKLTVDKSRW QQGNVFSCSVMHEALHNHYTQKSLSLSPGK |
| mRuby2-Fc | SRATMETDTLLLWVLLLWVPGSTGHHHHHHGGSENLYFQGGSVSKGEELIKEN MRMKVVMEGSVNGHQFKCTGEGEGNPYMGTQTMRIKVIEGGPLPFAFDILATS FMYGSRTFIKYPKGIPDFFKQSFPEGFTWERVTRYEDGGVVTVMQDTSLEDGC LVYHVQVRGVNFPSNGPVMQKKTKGWEPNTEMMYPADGGLRGYTHMALKVD GGGHLSCSFVTTYRSKKTVGNIKMPGIHAVDHRLERLEESDNEMFVVQREHAV AKFAGLGGGMDELYKGGSGSEPKSSDKTHTCPPCPAPELLGGPSVFLFPPKPK DTLMISRTPEVTCVVVDVSHEDPEVKFNWYVDGVEVHNAKTKPREEQYNSTYR VVSVLTVLHQDWLNGKEYKCKVSNKALPAPIEKTISKAKGQPREPQVYTLPPSR DELTKNQVSLTCLVKGFYPSDIAVEWESNGQPENNYKTTPPVLDSDGSFFLYSK LTVDKSRWQQGNVFSCSVMHEALHNHYTQKSLSLSPGK |
| Cetuximab light chain | MELGLSWIFLLAILKGVQCDILLTQSPVILSVSPGERVSFSCRASQSIGTNIHWYQ QRTNGSPRLLIKYASESISGIPSRFSGSGSGTDFTLSINSVESEDIADYYCQQNN NWPTTFGAGTKLELKRTVAAPSVFIFPPSDEQLKSGTASVVCLLNNFYPREAKV QWKVDNALQSGNSQESVTEQDSKDSTYSLSSTLTLSKADYEKHKVYACEVTHQ GLSSPVTKSFNRGEC |

| | |
|---|---|
| Cetuximab heavy chain | MELGLSWIFLLAILKGVQCQVQLKQSGPGLVQPSQSLSITCTVSGFSLTNYGVH WVRQSPGKGLEWLGVIWSGGNTDYNTPFTSRLSINKDNSKSQVFFKMNSLQS NDTAIYYCARALTYYDYEFAYWGQGTLVTVSAASTKGPSVFPLAPSSKSTSGGT AALGCLVKDYFPEPVTVSWNSGALTSGVHTFPAVLQSSGLYSLSSVVTVPSSSL GTQTYICNVNHKPSNTKVDKRVEPKSCDKTHTCPPCPAPELLGGPSVFLFPPKP KDTLMISRTPEVTCVVVDVSHEDPEVKFNWYVDGVEVHNAKTKPREEQYNSTY RVVSVLTVLHQDWLNGKEYKCKVSNKALPAPIEKTISKAKGQPREPQVYTLPPS REEMTKNQVSLTCLVKGFYPSDIAVEWESNGQPENNYKTTPPVLDSDGSFFLYS KLTVDKSRWQQGNVFSCSVMHEALHNHYTQKSLSLSPGKGSGHHHHHH |

**Supplementary Table 3: Amino acid sequences of molecular cargoes used by O432-17 designs.** “r” indicates RNA; “m” indicates 2' O-methyl RNA bases; “*” indicates a Phosphorothioate Bond.

| Design name | Sequence |
|---|---|
| pos36GFP | MGHHHHHHGGASKGERLFRGKVPILVELKGDVNGHKFSVRGKGKGDATRGKL TLKFICTTGKLPVPWPTLVTTLTYGVQCFSRYPKHMKRHDFFKSAMPKGYVQER TISFKKDGKYKTRAEVKFEGRTLVNRIKLKGRDFKEKGNILGHKLRYNFNSHKVY ITADKRKNGIKAKFKIRHNVKDGSVQLADHYQQNTPIGRGPVLLPRNHYLSTRSK LSKDPKEKRDHMVLLEFVTAAGIKHGRDERYK |
| pegRNA | mC*mC*mA*rGrGrCrUrUrCrCrGrGrGrUrCrArUrCrCrCrGrUrUrUrArGrArGrCrUr ArGrArArArUrArGrCrArArGrUrUrArArArArUrArArGrGrCrUrArGrUrCrCrGrUrUrAr UrCrArArCrUrUrGrArArArArArGrUrGrGrCrArCrCrGrArGrUrCrGrGrUrGrCrGrCrAr CrCrUrGrGrUrGrUrArUrGrArCrCrCrGrGrArCrGrCrGrGrUrUrCrUrArUrCrUrArGrU rUrArCrGrCrGrUrUrArArArCrCrArArCrUrA*mG*mA*mA |

**Supplementary Table 4: Details on EM data acquisition on different O432-17 samples**

| Sample name | Stain | Magnification | Pixel size (Å/pixel) | # Micrographs |
|---|---|---|---|---|
| O432-17 Fc | Uranyless | 45,000 | 3.16 | 289 |
| O432-17 CTX | Uranyless | 45,000 | 3.16 | 249 |
| O432-17(+) RNA CTX | Uranyless | 45,000 | 3.16 | 169 |
| O432-17(-) pos36GFP Fc | Uranyless | 45,000 | 3.16 | 289 |
| O432-17(-) | Uranyless | 45,000 | 3.14 | 215 |
| O432-17(-)_0HIS | Uranyless | 45,000 | 3.16 | 133 |
| O432-17(-)_3HIS_I57V | Uranyless | 45,000 | 3.14 | 174 |
| O432-17(-)_3HIS_L75A | Uranyless | 45,000 | 3.14 | 134 |

| | | | | |
|---|---|---|---|---|
| O432-17(-)_3HIS_I57V_L75A | Uranyless | 45,000 | 3.14 | 174 |
| O432-17(+) | Uranyless | 45,000 | 3.14 | 159 |
| O432-17(+)_0HIS | Uranyless | 45,000 | 3.14 | 148 |
| O432-17(+)_3HIS_I57V | Uranyless | 45,000 | 3.16 | 166 |
| O432-17(+)_3HIS_L75A | Uranyless | 45,000 | 3.14 | 234 |
| O432-17(+)_3HIS_I57V_L75A | Uranyless | 45,000 | 3.14 | 136 |

**Supplementary Table 5: Details on EM data processing on different O432-17 samples**

| Sample name | Particle picking | CTF estimation | 2D class averages | # particles in final selected 2D classes/total picked particles |
|---|---|---|---|---|
| O432-17 Fc | CisTEM | CTFFIND4 within Relion | Relion | 84107/112334 |
| O432-17 CTX | Relion template picking | CTFFIND4 within Relion | Relion | 23676/84107 |
| O432-17(+) RNA CTX | CryoSPARC template picking | CTFFIND4 within CryoSPARC | CryoSPARC | 18204/79682 |
| O432-17(-) pos36GFP Fc | CryoSPARC template picking | PatchCTF within CryoSPARC | CryoSPARC | 73011/174118 |
| O432-17(-) | CryoSPARC blob picking | PatchCTF within CryoSPARC | CryoSPARC | 39155/67832 |
| O432-17(-)_0HIS | CryoSPARC template picking | PatchCTF within CryoSPARC | CryoSPARC | 42476/100980 |
| O432-17(-)_3HIS_I57V | CryoSPARC template picking | PatchCTF within CryoSPARC | CryoSPARC | 24600/49412 |
| O432-17(-)_3HIS_L75A | CryoSPARC template picking | PatchCTF within CryoSPARC | CryoSPARC | 43698/90444 |
| O432-17(-)_3HIS_I57V_L75A | CryoSPARC blob picking | PatchCTF within CryoSPARC | CryoSPARC | 14002/27874 |

| | | | | |
|---|---|---|---|---|
| O432-17(+) | CryoSPARC blob picking | PatchCTF within CryoSPARC | CryoSPARC | 8286/17010 |
| O432-17(+)_0HIS | CryoSPARC blob picking | PatchCTF within CryoSPARC | CryoSPARC | 32809/112492 |
| O432-17(+)_3HIS_I57V | CryoSPARC blob picking | PatchCTF within CryoSPARC | CryoSPARC | 85680/125125 |
| O432-17(+)_3HIS_L75A | CryoSPARC template picking | PatchCTF within CryoSPARC | CryoSPARC | 25596/50100 |
| O432-17(+)_3HIS_I57V_L75A | CryoSPARC blob picking | PatchCTF within CryoSPARC | CryoSPARC | 15313/101228 |

**Supplementary Table 6: Statistical information for *in vitro* pH-mediated GFP release experiments (Fig. 4k).** All analyses were performed using Graphpad Prism version 9.3.1 Software.

| Fluorescent label | Tukey's multiple comparisons test | Mean Diff. | 95.00% CI of diff. | Below threshold? | Summary | Adjusted P Value |
|---|---|---|---|---|---|---|
| mRuby-Fc | O432-17(-) pH 8 vs. O432-17(-) pH 4 | -712.5 | -883.1 to -541.9 | Yes | **** | <0.0001 |
| | O432-17(-) pH 8 vs. O42.1 pH 8 | 88.33 | -82.30 to 259.0 | No | ns | 0.5896 |
| | O432-17(-) pH 8 vs. O42.1 pH 4 | -260.8 | -431.5 to -90.20 | Yes | *** | 0.0007 |
| | O432-17(-) pH 8 vs. Buffer | 133.5 | -37.14 to 304.1 | No | ns | 0.1914 |
| | O432-17(-) pH 4 vs. O42.1 pH 8 | 800.8 | 630.2 to 971.5 | Yes | **** | <0.0001 |
| | O432-17(-) pH 4 vs. O42.1 pH 4 | 451.7 | 281.0 to 622.3 | Yes | **** | <0.0001 |
| | O432-17(-) pH 4 vs. Buffer | 846 | 675.4 to 1017 | Yes | **** | <0.0001 |
| | O42.1 pH 8 vs. O42.1 pH 4 | -349.2 | -519.8 to -178.5 | Yes | **** | <0.0001 |
| | O42.1 pH 8 vs. Buffer | 45.17 | -125.5 to 215.8 | No | ns | 0.9437 |

| | | | | | | |
|---|---|---|---|---|---|---|
| | O42.1 pH 4 vs. Buffer | 394.3 | 223.7 to 565.0 | Yes | **** | <0.0001 |
| pos36GFP | O432-17(-) pH 8 vs. O432-17(-) pH 4 | -719.8 | -890.5 to -549.2 | Yes | **** | <0.0001 |
| | O432-17(-) pH 8 vs. O42.1 pH 8 | 41.67 | -129.0 to 212.3 | No | ns | 0.9575 |
| | O432-17(-) pH 8 vs. O42.1 pH 4 | -170.3 | -341.0 to 0.3025 | No | ns | 0.0506 |
| | O432-17(-) pH 8 vs. Buffer | -36.83 | -207.5 to 133.8 | No | ns | 0.9727 |
| | O432-17(-) pH 4 vs. O42.1 pH 8 | 761.5 | 590.9 to 932.1 | Yes | **** | <0.0001 |
| | O432-17(-) pH 4 vs. O42.1 pH 4 | 549.5 | 378.9 to 720.1 | Yes | **** | <0.0001 |
| | O432-17(-) pH 4 vs. Buffer | 683 | 512.4 to 853.6 | Yes | **** | <0.0001 |
| | O42.1 pH 8 vs. O42.1 pH 4 | -212 | -382.6 to -41.36 | Yes | ** | 0.008 |
| | O42.1 pH 8 vs. Buffer | -78.5 | -249.1 to 92.14 | No | ns | 0.6913 |
| | O42.1 pH 4 vs. Buffer | 133.5 | -37.14 to 304.1 | No | ns | 0.1914 |

**Supplementary Table 7: Statistical information for pH titration experiments.** All analyses were performed using Graphpad Prism version 9.3.1 Software.

| Experiment (Fig.) | Fluorescent label | Dunnett's multiple comparisons test | Mean Diff. | 95.00% CI of diff. | Below threshold ? | Summary | Adjusted P Value |
|---|---|---|---|---|---|---|---|
| Fig. 5c,d | AF647 | O432-17(-)_0HIS vs. O432-17(-)_2HIS | 0.06277 | -0.2448 to 0.3703 | No | ns | 0.9598 |
| Fig. 5d,e | AF647 | O432-17(-)_0HIS vs. O432-17(-)_3HIS_I57V | 0.25 | -0.05761 to 0.5575 | No | ns | 0.1407 |
| Fig. 5d,f | AF647 | O432-17(-)_0HIS vs. O432-17(-)_3HIS_I5 | 0.388 | 0.08047 to 0.6956 | Yes | ** | 0.0092 |

| | | | | | | | |
|---|---|---|---|---|---|---|---|
| | | 7V_L75A | | | | | |
| Fig. 5d,g | AF647 | O432-17(-)_0HIS vs. O432-17(-)_3HIS_74A | 0.1996 | -0.1080 to 0.5071 | No | ns | 0.3019 |
| Fig. 5c,d | sfGFP | O432-17(-)_0HIS vs. O432-17(-)_2HIS | 0.02994 | -0.2523 to 0.3122 | No | ns | 0.9964 |
| Fig. 5d,e | sfGFP | O432-17(-)_0HIS vs. O432-17(-)_3HIS_I57V | 0.1138 | -0.1684 to 0.3960 | No | ns | 0.6984 |
| Fig. 5d,f | sfGFP | O432-17(-)_0HIS vs. O432-17(-)_3HIS_I57V_L75A | 0.07698 | -0.2052 to 0.3592 | No | ns | 0.8962 |
| Fig. 5d,g | sfGFP | O432-17(-)_0HIS vs. O432-17(-)_3HIS_L75A | -0.02741 | -0.3096 to 0.2548 | No | ns | 0.9974 |
| Supplementary Fig. 8e | AF647 | O432-17(+)_0HIS vs. O432-17(+)_3HIS_I57V | 0.3731 | 0.04387 to 0.7024 | Yes | * | 0.0231 |
| | | O432-17(+)_0HIS vs. O432-17(+)_3HIS_I57V_L75A | 0.3502 | 0.02095 to 0.6794 | Yes | * | 0.0349 |
| | | O432-17(+)_0HIS vs. O432-17(+)_3HIS_L75A | 0.3564 | 0.02720 to 0.6857 | Yes | * | 0.0312 |
| Supplementary Fig. 8f | sfGFP | O432-17(+)_0HIS vs. O432-17(+)_3HIS_I57V | 0.3094 | -0.02647 to 0.6454 | No | ns | 0.0766 |
| | | O432-17(+)_0HIS vs. O432-17(+)_3HIS_I57V_L75A | 0.3221 | -0.01386 to 0.6580 | No | ns | 0.0627 |
| | | O432-17(+)_0HIS vs. O432-17(+)_3HIS_L75A | 0.2852 | -0.05070 to 0.6211 | No | ns | 0.1109 |

**Supplementary Table 8: Statistical information for uptake of O432-17-CTX and O432-17-Fc nanoparticles in eukaryotic cells.** All analyses were performed using Graphpad Prism version 9.3.1 Software.

| Experiment (Fig.) | Comparison | Test | Adjusted P Value |
|---|---|---|---|
| Supplementary Fig. 10c | % A431 cells with cage (O432-17-CTX vs. O432-17-Fc) | Two-tailed Unpaired T Test | 0.0306 |
| Supplementary Fig. 10d | Intensity of cages in A431 cells (O432-17-CTX vs. O432-17-Fc) | Two-tailed Unpaired T Test | 0.6025 |
| Supplementary Fig. 10f | HeLa WT with serum vs. HeLa WT without serum | One-way ANOVA with Tukey's correction for multiple comparisons | 0.0116 |
| | HeLa WT with serum vs. HeLa EGFR KO with serum | One-way ANOVA with Tukey's correction for multiple comparisons | 0.001 |
| | HeLa WT without serum vs. HeLa EGFR KO without serum | One-way ANOVA with Tukey's correction for multiple comparisons | <0.0001 |
| | HeLa EGFR KO with serum vs. HeLa EGFR KO without serum | One-way ANOVA with Tukey's correction for multiple comparisons | 0.9349 |
| Supplementary Fig. 10g | HeLa WT with serum vs. HeLa WT without serum | One-way ANOVA with Tukey's correction for multiple comparisons | 0.9598 |
| | HeLa WT with serum vs. HeLa EGFR KO with serum | One-way ANOVA with Tukey's correction for multiple comparisons | 0.9298 |
| | HeLa WT without serum vs. HeLa EGFR KO without serum | One-way ANOVA with Tukey's correction for multiple comparisons | 0.0359 |
| | HeLa EGFR KO with serum vs. HeLa EGFR KO without serum | One-way ANOVA with Tukey's correction for multiple comparisons | 0.0161 |

**Supplementary Table 9: AF647-Nanoparticle conjugation efficiencies and related values.**

| Sample | AF647 Conjugation Efficiency % | Number of AF647 fluorophores per nanoparticle |
|---|---|---|

| | | |
|---|---|---|
| O432-17(-)_2HIS-C3 | 75% | 18 |
| O432-17(-)_3HIS_I57V_L75A-C3 | 21% | 5 |
| O432-17(-)_0HIS-C3 | 63% | 15 |
| O432-17(-)_3HIS_I57V-C3 | 37% | 9 |
| O432-17(-)_3HIS_L75A-C3 | 30% | 7 |
| O432-17(+)_2HIS-C3 | 21% | 5 |
| O432-17(+)_3HIS_I57V-C3 | 62% | 15 |
| O432-17(+)_3HIS_L75A-C3 | 65% | 16 |
| O432-17(+)_3HIS_I57V_L75A-C3 | 61% | 15 |
| O432-17(+)_0HIS-C3 | 72% | 17 |

# Supplementary Figures

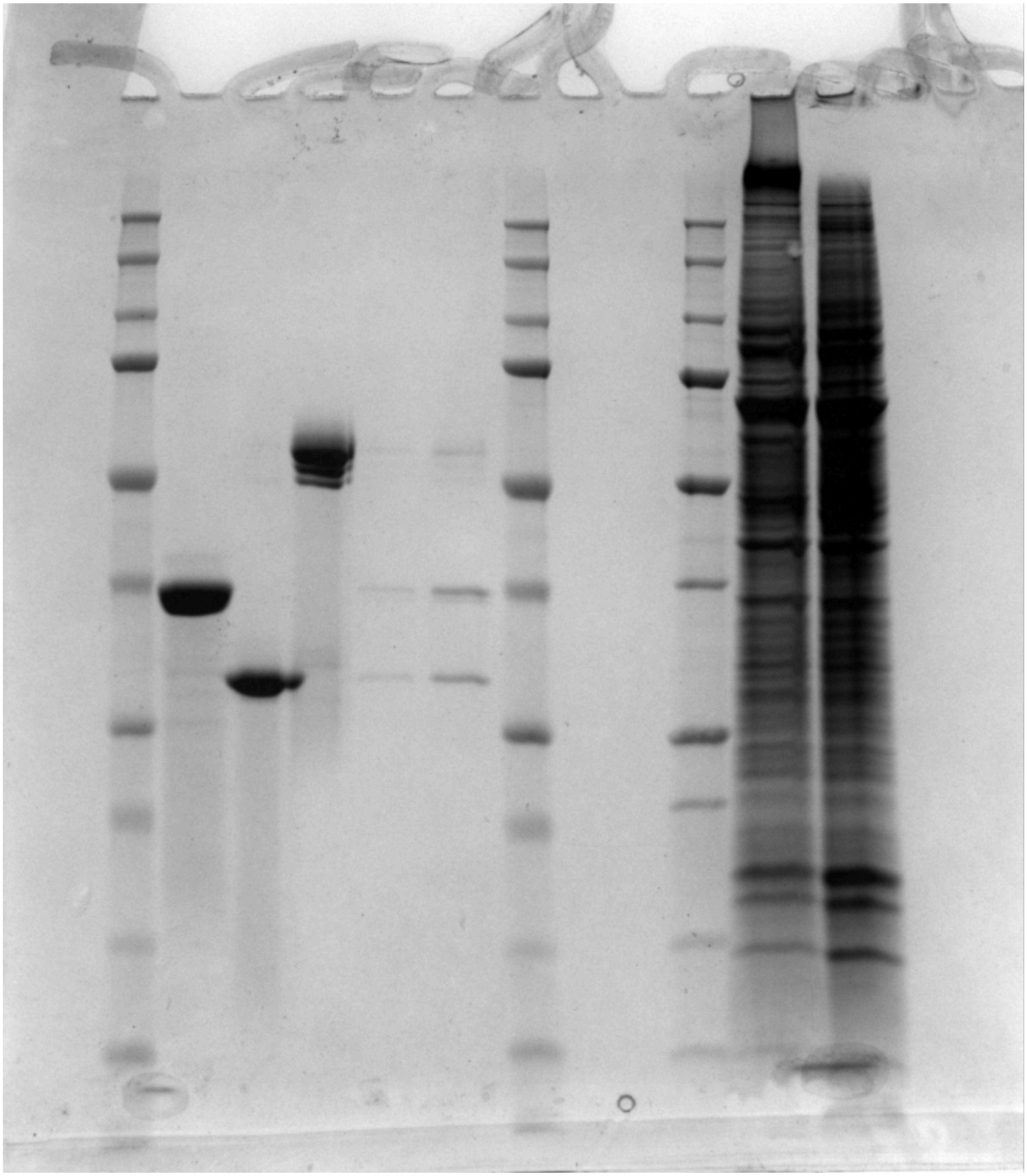

**Supplementary Figure 1**: Uncropped and unprocessed gel for Supplementary Figure 3e.

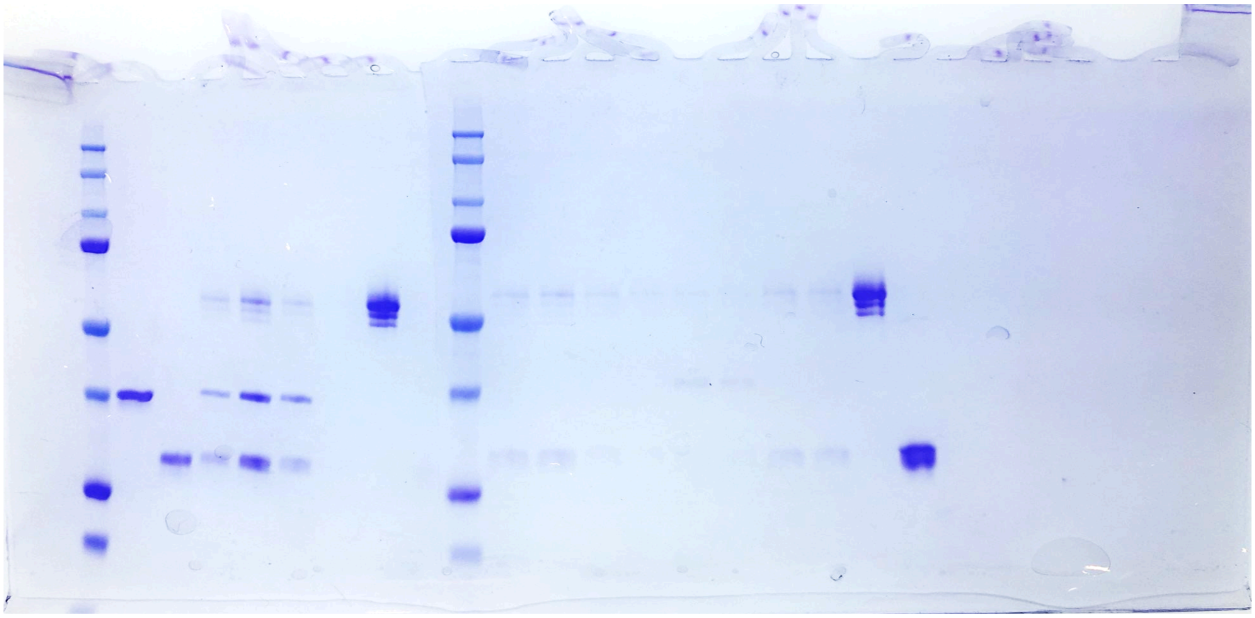

**Supplementary Figure 2**: Uncropped and unprocessed gel for Supplementary Figure 3f.

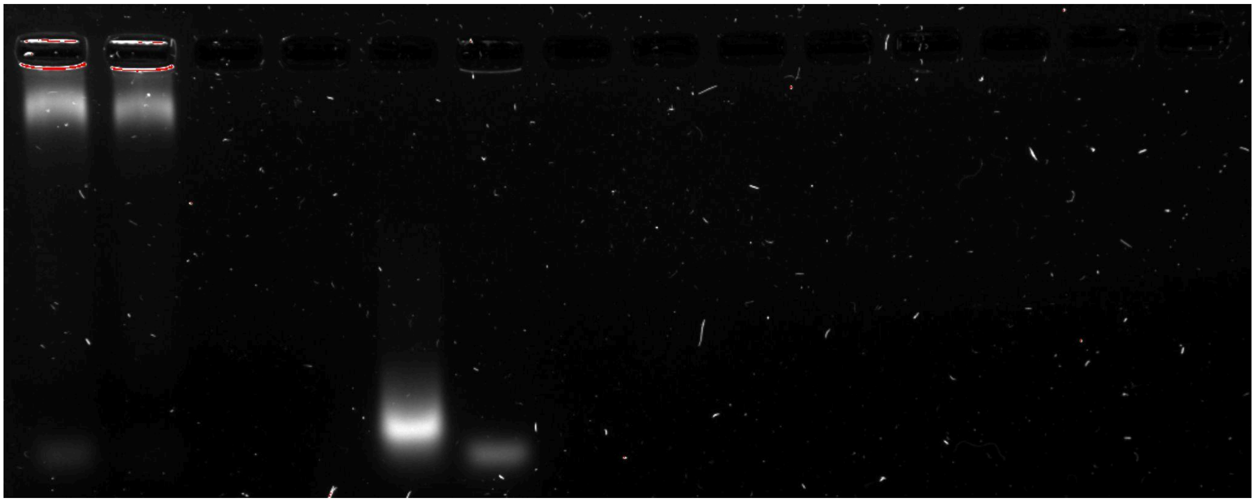

**Supplementary Figure 3**: Uncropped and unprocessed gel for Supplementary Figure 9e.

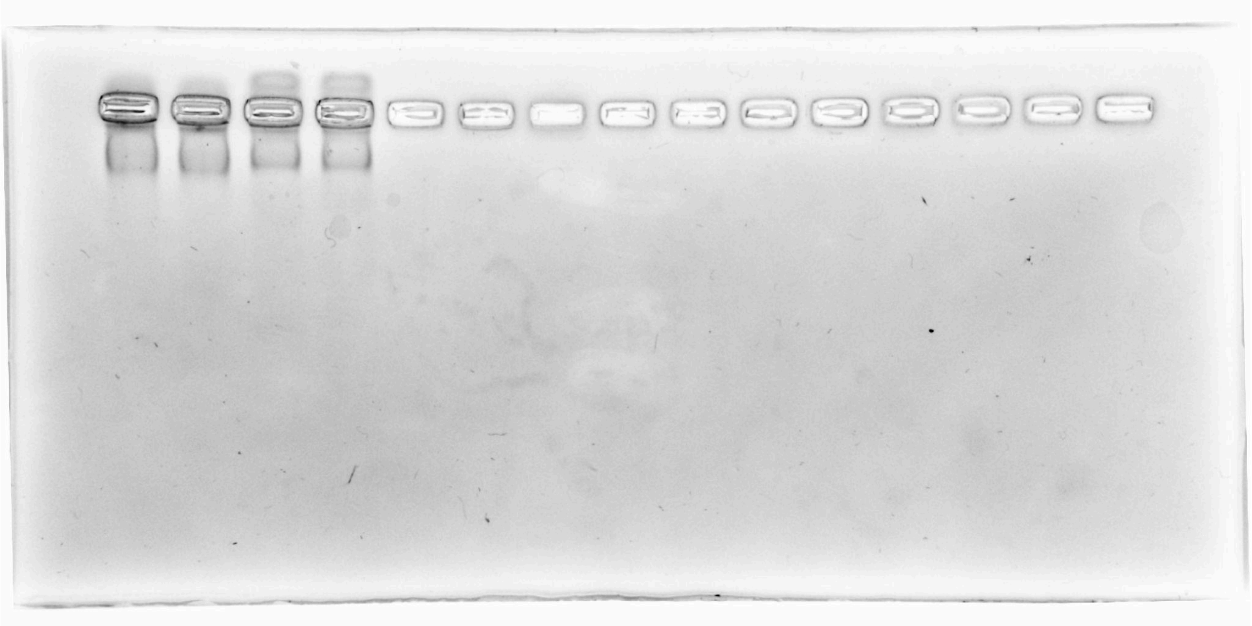

**Supplementary Figure 4**: Uncropped and unprocessed gel for Supplementary Figure 9f.
