# Supplementary material for: Computational design of non-porous pH-responsive antibody nanoparticles
Source: Nat Struct Mol Biol. 2024 May 9;31(9):1404–12. doi: 10.1038/s41594-024-01288-5 (PMC11402598; doi:10.1038/s41594-024-01288-5)
Supplement: Supplementary file 2 — Reporting Summary [file 41594_2024_1288_MOESM2_ESM.pdf]

nature portfolio

Corresponding author(s): David Baker

Last updated by author(s): Dec 5, 2023

Reporting Summary

Nature Portfolio wishes to improve the reproducibility of the work that we publish. This form provides structure for consistency and transparency in reporting. For further information on Nature Portfolio policies, see our Editorial Policies and the Editorial Policy Checklist.

Statistics

For all statistical analyses, confirm that the following items are present in the figure legend, table legend, main text, or Methods section.

n/a	Confirmed	
☐	☒	The exact sample size (n) for each experimental group/condition, given as a discrete number and unit of measurement
☐	☒	A statement on whether measurements were taken from distinct samples or whether the same sample was measured repeatedly
☐	☒	The statistical test(s) used AND whether they are one- or two-sided<br>*Only common tests should be described solely by name; describe more complex techniques in the Methods section.*
☐	☒	A description of all covariates tested
☐	☒	A description of any assumptions or corrections, such as tests of normality and adjustment for multiple comparisons
☐	☒	A full description of the statistical parameters including central tendency (e.g. means) or other basic estimates (e.g. regression coefficient) AND variation (e.g. standard deviation) or associated estimates of uncertainty (e.g. confidence intervals)
☐	☒	For null hypothesis testing, the test statistic (e.g. F, t, r) with confidence intervals, effect sizes, degrees of freedom and P value noted<br>*Give P values as exact values whenever suitable.*
☒	☐	For Bayesian analysis, information on the choice of priors and Markov chain Monte Carlo settings
☒	☐	For hierarchical and complex designs, identification of the appropriate level for tests and full reporting of outcomes
☒	☐	Estimates of effect sizes (e.g. Cohen's d, Pearson's r), indicating how they were calculated

Our web collection on statistics for biologists contains articles on many of the points above.

Software and code

Policy information about availability of computer code

Data collection	Both the worms (https://github.com/willsheffler/worms) and RPXDock (https://github.com/willsheffler/rpxdock) software codes are available publicly on GitHub. Code was developed as part of this project and provided in separate publications; there are no version accession numbers for these repositories. An example design protocol used to generate O432-17 nanoparticles is provided in the following github link: https://github.com/erincyang/plug_design<br>Electron microscopy processing was done using Relion 3.1 or CryoSPARC4.2. Particles for Fig 1D (top) were picked with CisTEM and imported into Relion for subsequent 2D averaging<br>Images were created in UCSF ChimeraX 1.6 and the PyMOL Molecular Graphics System, version 2.5 (Schrödinger), and Biorender
Data analysis	Flow cytometry analyses were performed in Flowjo, LLC softwarev10.10<br>Statistical analyses were performed in GraphPad Prism v9.3.1<br>Cell images were analyzed in ImageJ v2.<br>Electron microscopy analysis was performed by CryoSPARC software v4.0.3 or Relion3.0 software

For manuscripts utilizing custom algorithms or software that are central to the research but not yet described in published literature, software must be made available to editors and reviewers. We strongly encourage code deposition in a community repository (e.g. GitHub). See the Nature Portfolio guidelines for submitting code & software for further information.

Data

Policy information about availability of data

All manuscripts must include a data availability statement. This statement should provide the following information, where applicable:

- Accession codes, unique identifiers, or web links for publicly available datasets
- A description of any restrictions on data availability
- For clinical datasets or third party data, please ensure that the statement adheres to our policy

Source data for all images and data generated and analyzed by the authors are provided with this manuscript. Density maps have been deposited in the Electron Microscopy Data Bank under the accession number EMD-29602.

Human research participants

Policy information about studies involving human research participants and Sex and Gender in Research.

Reporting on sex and gender	N/A
Population characteristics	N/A
Recruitment	N/A
Ethics oversight	N/A

Note that full information on the approval of the study protocol must also be provided in the manuscript.

Field-specific reporting

Please select the one below that is the best fit for your research. If you are not sure, read the appropriate sections before making your selection.

☒ Life sciences ☐ Behavioural & social sciences ☐ Ecological, evolutionary & environmental sciences

For a reference copy of the document with all sections, see nature.com/documents/nr-reporting-summary-flat.pdf

Life sciences study design

All studies must disclose on these points even when the disclosure is negative.

Sample size	In vitro release experiments were performed with three biologically independent replicates and measured in duplicate. All cell uptake experiments were performed in duplicate. Three images were acquired per condition and analyses were carried out for all cells in each image.
Data exclusions	Outliers with low bead or cell counts were excluded from the data analysis in the flow cytometry data.
Replication	Details on electron microscopy experiments can be found in Supplementary Table 4. All gel electrophoresis experiments were replicated in at least two biologically independent experiments. All experiments were executed successfully. Electron micrographs with poor CTF estimations were excluded from data analysis.
Randomization	Cell and bead experiments were randomized such that replicate within a condition received a biased subset of cells
Blinding	All cell and bead experiments were performed with blinding study design

Reporting for specific materials, systems and methods

We require information from authors about some types of materials, experimental systems and methods used in many studies. Here, indicate whether each material, system or method listed is relevant to your study. If you are not sure if a list item applies to your research, read the appropriate section before selecting a response.

Materials & experimental systems

n/a	Involved in the study
☐	☒ Antibodies
☐	☒ Eukaryotic cell lines
☒	☐ Palaeontology and archaeology
☒	☐ Animals and other organisms
☒	☐ Clinical data
☒	☐ Dual use research of concern

Methods

n/a	Involved in the study
☒	☐ ChIP-seq
☐	☒ Flow cytometry
☒	☐ MRI-based neuroimaging

Antibodies

Antibodies used	Myc-Tag (9B11) Mouse mAb (Cell Signaling Technologies, #2276), Anti-LAMP2A antibody (Abcam ab18528, dilution: 1:200), goat anti-rabbit- IgG Alexa Fluor™ 488 secondary antibody (Thermo Fisher A-11034, dilution 1:200), Cetuximab (Table 3, Methods), Anti-NaK ATPase (Abcam ab76020, Abcam ab283318, dilution: 1:200x), goat anti-mouse-IgG Alexa Fluor™ 488 secondary antibody (Thermo Fisher A-11029, dilution: 1:200x)
Validation	Commercial antibodies validated by manufacturers and validated via a certificate of analysis with lot number, measured concentration, and approved applications for Flow Cytometry and Immunoprecipitation<br>Cetuximab antibody was produced in house following the materials and methods protocol and SEC and assembly data validation is shown in the manuscript (Figure 2)

Eukaryotic cell lines

Policy information about cell lines and Sex and Gender in Research

Cell line source(s)	All cell lines are reported in materials and methods. ExpiHEK293F (Thermo Fisher A14527), WT HeLa (ATCC CCL-2), EGFR KO HeLa (Abcam ab255385), and A431 cells (ATCC CRL-1555)
Authentication	Authentication was performed by the manufacturer for growth rate, mycoplasma contamination, STR profiling, and cell viability, and tested routinely during culturing for cell viability, cell morphology, and mycoplasma contamination.
Mycoplasma contamination	Cell lines tested negative for mycoplasma contamination upon thawing and were tested routinely during culturing for negative mycoplasma contamination
Commonly misidentified lines (See ICLAC register)	No commonly misidentified lines were used in this study.

Flow Cytometry

Plots

Confirm that:

☒ The axis labels state the marker and fluorochrome used (e.g. CD4-FITC).

☒ The axis scales are clearly visible. Include numbers along axes only for bottom left plot of group (a 'group' is an analysis of identical markers).

☒ All plots are contour plots with outliers or pseudocolor plots.

☒ A numerical value for number of cells or percentage (with statistics) is provided.

Methodology

Sample preparation	Linear myc peptide with an N terminal lysine side chain and 3× glycine linker (KGGGEQKLISEEDL) was produced via solid-phase peptide synthesis and biotinylated via amide formation. The resulting biotinylated myc peptide was purified by RP-HPLC and quality checked for the proper molecular weight via LC-MS, lyophilized, and dissolved in 100% DMSO for long term storage. 3.0-3.4 µm streptavidin coated polystyrene particles (Spherotech) were incubated with biotinylated myc peptide diluted in PBS to 5% DMSO for 20 minutes at 25°C. Following two washes in PBS + 3% BSA by centrifugation at 3000×G for 5 minutes, coated polystyrene particles were incubated with α-myc-O432-17 nanoparticle variants for one hour at 25°C. α-myc antibody was purchased from Cell Signaling Technologies (9B11). The coated particles were washed in PBS + 3% BSA twice and split equally into pH-titrated citrate-phosphate buffers for 30 minutes at 25°C. Coated particles were washed twice with PBS + 3% BSA and resuspended for flow-cytometry. For O432-17(+) experiments, we prevented non-specific association to the polystyrene particles by substituting with EBY100 S. cerevisiae cells.<br><br>All flow-cytometry experiments were performed on a LSR II Flow Cytometer (BD Biosciences). Lasers were calibrated with coated particles stained with either FITC Anti-Myc tag antibody (Abcam 9E10), APC Anti-Myc tag antibody (Abcam 9E10), or no antibody. 10,000 events were collected per sample on three biological replicates. All flow-cytometry results were analyzed using the FlowJo, LLC software. Singlet beads were first isolated before analyzing for AF647 or sfGFP signal. Normalization to the minimum and maximum fluorescence of each channel with each titration sample was performed in Python3.7, and the

apparent pKa of AF647 and sfGFP fluorescence was estimated with a four parameter nonlinear logistic regression fit in GraphPad Prism version 9.3.1 for Windows, GraphPad Software, San Diego, California USA, www.graphpad.com.

Instrument	BD LSR II Flow Cytometer
Software	Flowjov10.8
Cell population abundance	Cell population abundance is shown in the supplementary materials (Supplementary Figure 8). 10,000 events were collected per well. Approximately 80% of the bead population was gated for bead morphology and purity, nearly all gated beads (99%) were singlet beads which were analyzed for Alexa-647 and FITC fluorescence.
Gating strategy	The example gating strategy is shown in Supplementary Figure 8. Beads were initially gated on SSC-A and FSC-A, identifying a dense population of beads without abnormal morphologies or contamination. Singlet beads were identified using FSC-H and FSC-A. The cutoff for positive Alexa 647-A fluorescence was determined using the negative control for beads incubated with Alexa-647-conjugated trimer. The FITC cutoff was determined using a negative control for beads incubated with sfGFP-Fc.

☒ Tick this box to confirm that a figure exemplifying the gating strategy is provided in the Supplementary Information.
