# Supplementary figures and images for: Computational design of non-porous pH-responsive antibody nanoparticles
Source: Nat Struct Mol Biol. 2024 May 9;31(9):1404–12. doi: 10.1038/s41594-024-01288-5 (PMC11402598; doi:10.1038/s41594-024-01288-5)

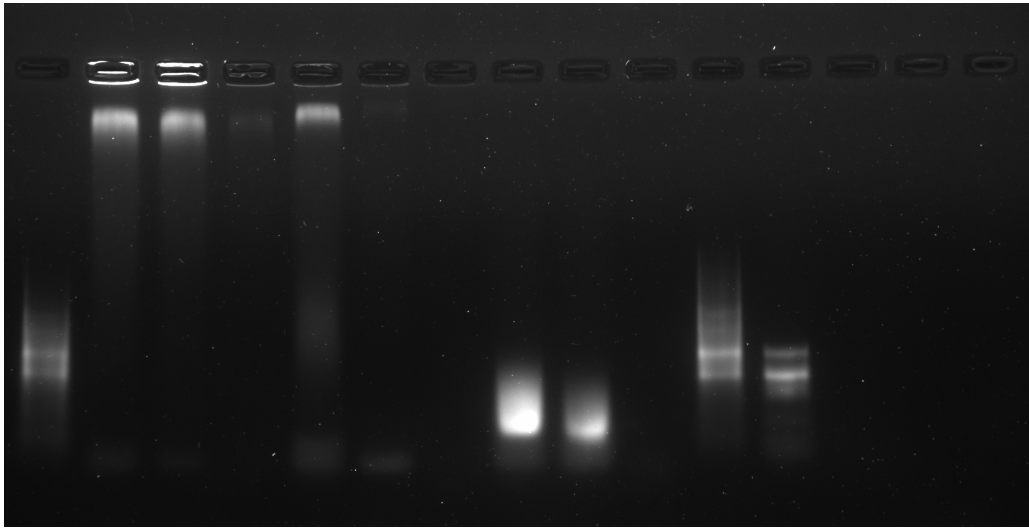

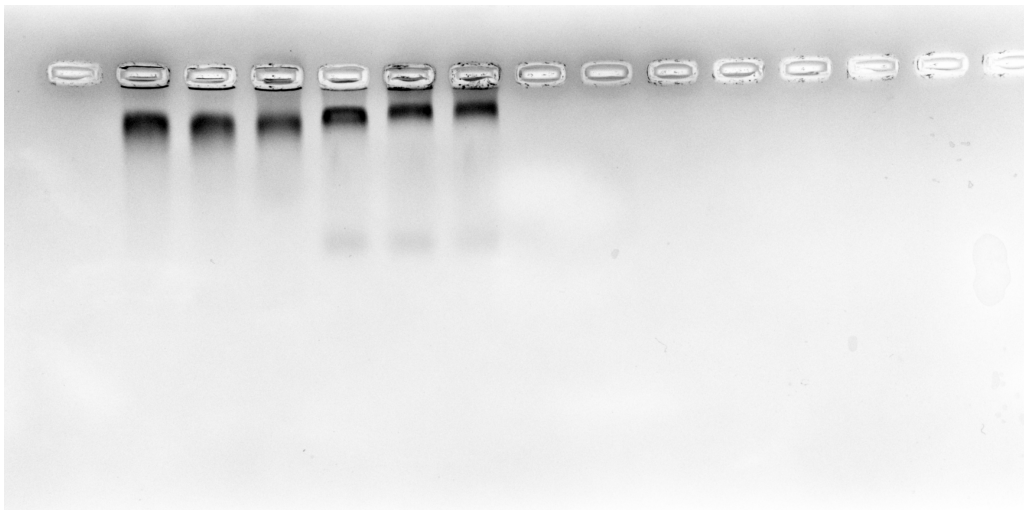

Supplement: Supplementary file 6 — Umodified gels. [file 41594_2024_1288_MOESM6_ESM.pdf]
